# The Pharmacological Effect and Mechanism of Lanthanum Hydroxide on Vascular Calcification Caused by Chronic Renal Failure Hyperphosphatemia

**DOI:** 10.3389/fcell.2021.639127

**Published:** 2021-04-13

**Authors:** Lulu Zhao, Shengnan Wang, Hong Liu, Xiaoli Du, Ren Bu, Bing Li, Ruilan Han, Jie Gao, Yang Liu, Jian Hao, Jianrong Zhao, Yan Meng, Gang Li

**Affiliations:** ^1^Department of Pharmacology, College of Pharmacy, Inner Mongolia Medical University, Jinshan Development, Hohhot, China; ^2^Department of Nephrology, The Affiliated Hospital of Inner Mongolia Medical University, Hohhot, China

**Keywords:** lanthanum hydroxide, vascular calcification, chronic renal failure, hyperphosphatemia, pharmacological effect, mechanism

## Abstract

**Objective:**

The present work aimed to explore the efficacy of lanthanum hydroxide in managing the vascular calcification induced by hyperphosphate in chronic renal failure (CRF) as well as the underlying mechanism.

**Methods:**

Rats were randomly allocated to five groups: normal diet control, CKD hyperphosphatemia model, CKD model treated with lanthanum hydroxide, CKD model receiving lanthanum carbonate treatment, together with CKD model receiving calcium carbonate treatment. The serum biochemical and kidney histopathological parameters were analyzed. The aortic vessels were subjected to Von Kossa staining, CT scan and proteomic analysis. *In vitro*, the calcium content and ALP activity were measured, and RT-PCR (SM22α, Runx2, BMP-2, and TRAF6) and Western blot (SM22α, Runx2, BMP-2, TRAF6, and NF-κB) were performed.

**Results:**

In the lanthanum hydroxide group, serum biochemical and kidney histopathological parameters were significantly improved compared with the model group, indicating the efficacy of lanthanum hydroxide in postponing CRF progression and in protecting renal function. In addition, applying lanthanum hydroxide postponed hyperphosphatemia-mediated vascular calcification in CKD. Furthermore, lanthanum hydroxide was found to mitigate vascular calcification via the NF-κB signal transduction pathway. For the cultured VSMCs, lanthanum chloride (LaCl_3_) alleviated phosphate-mediated calcification and suppressed the activation of NF-κB as well as osteo-/chondrogenic signal transduction. Lanthanum hydroxide evidently downregulated NF-κB, BMP-2, Runx2, and TRAF6 expression.

**Conclusion:**

Lanthanum hydroxide protects against renal failure and reduces the phosphorus level in serum to postpone vascular calcification progression.

## Introduction

Chronic kidney disease (CKD) refers to any abnormality affecting the renal structure and function that lasts for over 3 months (Inker et al., 2014). As reported in a recent systematic analysis, approximately 10.4% of the male and 11.8% of the female adult populations in the world were affected by CKD in 2010, and the incidence is comparatively high among the low/middle-income areas, while low among the developed countries (Mills et al., 2015).

Cardiovascular diseases often result in other comorbidities such as CKD, which may lead to CKD-related death, and this occurs in 30–50% of all CKD-related as well as end-stage renal disease (ESRD)-related deaths globally (Collins et al., 2015; Masakane et al., 2015). Vascular calcification has been identified as the independent risk factor that increases CVD deaths among CKD cases. Many studies have confirmed that hyperphosphatemia can cause cardiovascular complications, such as vascular calcification and left ventricular hypertrophy, thereby increasing the risk of death from CVD in CKD patients (Goodman et al., 2000; Block et al., 2004; Russo et al., 2011; Briet and Burns, 2012; Smith, 2016).

There are four types of vascular calcification: arterial intima calcification, arterial media calcification, aortic valve calcification and calciphylaxis. Arterial intima calcification and media calcification occur in ESRD patients, with intima calcification being the most frequent type (Wang et al., 2001). Therefore, intima and media calcification may be the main reason for the increased risk (10 to 100 fold) of cardiovascular death in patients with ESRD by 10 to 100 times (Huveneers et al., 2015). Intima calcification and media calcification may simultaneously exist in ESRD patients, but their manifestations may be very different. Intima calcification is confined to plaques, and the pathological changes are related to inflammatory reactions and lipid deposition. The main consequences may be plaque rupture and acute vascular occlusion, which may lead to avascular necrosis. Media calcification, which is also called the Monckeberg’s sclerosis, usually induces disorders within the inner elastic layer, which is manifested by linear deposition of hydroxyapatite calcium crystals in the inner elastic layer of the arterial media. Compared with intima calcification, there are few inflammatory cells or lipid deposition. The disorder mainly causes arterial stiffness and increased systolic blood pressure, leading to left ventricular hypertrophy, which in turn leads to heart failure. Studies have shown that calcification in the middle layer of arteries is a vital factor for predicting CVD-related mortality among ESRD cases (London et al., 2003).

Among the various causes of vascular calcification in CKD patients, hyperphosphatemia is closely related to vascular calcification. The absorption and excretion of phosphorus by the intestines, renal tubules, intracellular and extracellular fluids, and bones maintains the serum phosphorus levels within the normal range. Among them, the kidney’s filtration and absorption of phosphorus are the main factors affecting the steady state of serum phosphorus.

In CKD stage 2–3, the nephron weight decreases and the renal function declines. Phosphate hormones, including fibroblast growth factor 23 (FGF 23) or parathyroid hormone (PTH), are synthesized to resist excessive phosphorus. Such hormones play a major role in the renal proximal tubules, by downregulating type IIa and type IIc sodium-phosphorus transporters, thereby increasing renal phosphorus excretion and maintaining serum phosphorus levels within the normal range (Isakova et al., 2011; Tatsumi et al., 2016). However, as CKD enters the advanced stage, the kidneys can no longer effectively filter the ingested phosphorus, which eventually leads to obvious hyperphosphatemia in CKD stage 4–5. Hyperphosphatemia may lead to the significantly increased morbidity and mortality of vascular calcification, as seen in CKD cases and the general population (Dhingra et al., 2007; Da et al., 2015).

According to the current research on the mechanism of vascular calcification, effective control of serum phosphorus levels has a certain protective effect on the cardiovascular system and kidneys in patients with chronic kidney disease, especially in advanced patients. However, even after, controlling the intake of high-phosphorus foods and administering adequate dialysis, blood phosphorus levels may still not be effectively controlled within the ideal range. The use of phosphorus binders has become an important method to control phosphorus in the blood, mainly by combining with phosphorus in food to reduce phosphorus absorption. Oral phosphate binders combine with dietary phosphates in the intestines to prevent phosphorus absorption and become an important part of the management strategy to decrease and control the level of serum phosphorus in patients with advanced CKD. Some observational research data indicate that the use of phosphate adhesives can prolong the survival time of patients undergoing hemodialysis.

In the current treatment of hyperphosphatemia binders, lanthanum drugs have obvious advantages. Trivalent cations have a high affinity with phosphorus and are easy to form poorly soluble complexes with phosphorus. They are poorly soluble in low water conditions, and it is difficult to pass through the intestinal wall into the blood. They can be excreted through feces to achieve the effect of reducing phosphorus. However, lanthanum does not affect the absorption of fat-soluble vitamins and has no obvious side effects. Lanthanum absorbed into the body is mainly excreted through the liver, and therefore, it is suitable for dialysis patients. In the preliminary research of the research group, it was found that nano-lanthanum hydroxide can satisfactorily reduce phosphorus and also has a protective effect on the kidneys (Ma et al., 2019). The present work aimed to explore the clinical efficacy of lanthanum hydroxide in treating vascular calcification induced by hyperphosphate in the context of chronic renal failure (CRF).

## Materials and Methods

### Reagents and Antibodies

Lanthanum hydroxide (Laboratory synthesis), Adenine (Sigma-Aldrich, Cat. #V900471, United States), Lanthanum carbonate (Sigma-Aldrich, Cat. #325767 United States), Calcium carbonate (Sigma-Aldrich, Cat. #V900138 United States), Human aortic vascular smooth muscle cells (Shanghai Yubo Biological Technology Co., Ltd., Cat. #C740, China), RNA Simple Total RNA Kit (Tiangen Biochemical Technology (Beijing) Co., Ltd., Cat. #DP419, China), ReverTra Ace qPCR RT Kit (ToYoBo Life Science, Cat. #FSQ-101, China), SYBR Green Realtime PCR Master Mix (ToYoBo Life Science, Cat. #QPK-201), Lipopolysaccharide (LPS, Sigma-Aldrich, Cat. #L4391), Lanthanum chloride (LaCl3, Sigma-Aldrich, Cat. #298182), Sodium pyrophosphate tetrabasic decahydrate (PPI, Sigma-Aldrich, Cat. #S6422), Monoclonal Mouse anti-SM22α (SC-53932, Santa Cruz Biotechnology, RRID: AB_1129519), Monoclonal Mouse anti-BMP-2/4 (SC-137087, Santa Cruz Biotechnology, RRID: AB_2258985), Monoclonal Mouse anti-Runx2 (SC-101145, Santa Cruz Biotechnology, RRID: AB_1128251), Polyclonal Rabbit anti-β-Actin (K101527P, Solarbio), Monoclonal Rabbit anti-TRAF6 (ab40675, Abcam, RRID: AB_778573), Monoclonal Mouse anti-NF-κB (#6956, Cell Signaling Technology, RRID: AB_10828935), Goat anti-Mouse IgG (H + L) (A23910, Abbkine), Goat anti-Rabbit IgG (H + L) (A23920, Abbkine), Polyclonal Rabbit anti-Lamin A (10298-1-AP, Proteintech, RRID:AB_2296961).

### Animals and Experimental Protocol

The 6-week-old male wistar rats weighing 200–220 g (SPF, Beijing Vital River Laboratory Animal Technology Co., Ltd., China) were housed under the barrier environment, with constant temperature (21–22°C) and humidity (40–50%). All animals assigned in this study were given free access to their designed diet and tap water. Each rat was acclimatized to the experimental conditions for 1 week ahead of time.

**FIGURE 1:**
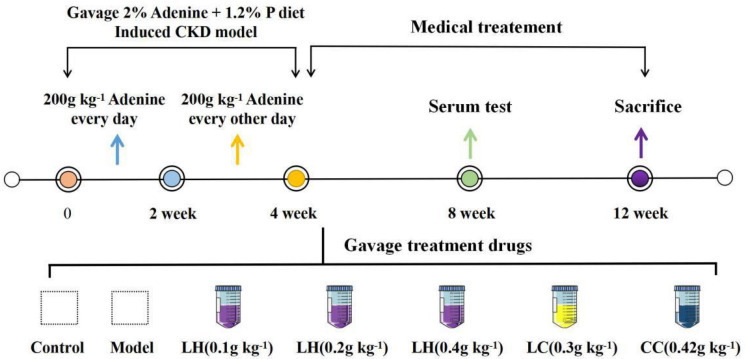
Protocols for the induction of CKD and phosphate loading. Except the control group, all other groups were 1.2% high phosphorus diet for 12 weeks.

The CKD animal model was established as previously described in Figure 1. For 1–2 weeks, the model rats were given 2% adenine gavaged at 200 mg kg^–1^ per day. For 3–4 weeks, all model rats gavaged every other day at the same concentration and dose. After 4 weeks, blood was collected from the fundus venous plexus of the rats, and serum phosphorus, creatinine, and urea nitrogen were detected to determine whether the model was successful. After the CKD rats were successful, the model group is subdivided into Lanthanum hydroxide (0.4 g kg^–1^, 0.2 g kg^–1^, and 0.1 g kg^–1^), lanthanum carbonate (0.3 g kg^–1^), calcium carbonate (4.2 g kg^–1^) and model group (*n* = 12), which was gavaged every day for 8 weeks. During the experimental period, rats were fed with standard chow in the control group or chow containing 1.2% phosphorus (Beijing Keao Xieli Feed Co., Ltd) in the CKD group. Serum phosphorus, creatinine, urea-nitrogen, PTH, FGF23 was measured at 4 and 8 weeks after treatment. On the last day of the 12 weeks, all animals were sacrificed. One day before sacrifice, the 12 h urine samples were collected from rats that were allowed to drink water only. To sacrifice the rats, each animal was injected with 50 mg kg^–1^ pentobarbital for anesthesia, and blood was collected from the abdominal aorta. Thereafter, aorta of every animal was collected and processed below: snap-frozen within liquid nitrogen to carry out RNA analysis and Western blotting assay, right kidney tissues were immersed into 10% phosphate-buffered formalin to conduct histological analysis. Besides, we separate the femoral bone, removed the soft tissues on it and then immersed it into the 10% phosphate-buffered formalin to perform histological analysis. Animal experiments were approved by the Medical Ethics Committee of Inner Mongolia Medical University (Document NO. YKD2019019).

### Cell Culture

The human aortic vascular smooth muscle cells (VSMCs) were cultured within the high-glucose (HG) DMEM (Gibco, Cat. #11995-065, United States) that contained 10% fetal bovine serum (FBS, Gibco, Cat. #10099-141, United States) as well as 1% penicillin-streptomycin (Gibco, Cat. #15140122, United States), and this medium was called the growth medium (GM). After achieving cell confluence, cells at passages 5–7 were used in our experiments. The medium was replaced at intervals of 2 days. For the sake of inducing phosphate-induced calcification, the 3 mmol/L inorganic phosphate (namely, the calcification medium, Na_2_HPO_4_/NaH_2_PO_4_, pH 7.4) was added into GM with or without La (15–60 μmol L^–1^ of LaCl_3_ × 6H_2_O) for a period of 6 days.

### Serum Biochemistry

Serum calcium, phosphorus, creatinine, and urea levels were measured using biochemical detection kit (Nanjing Jiancheng Bioengineering Institute, China). The Urease-GLDH approach was used to evaluate urea content. The sarcosine oxidase approach was used to measure creatinine content, whereas the MTB approach was used to evaluate calcium content. PTH and FGF23, were measured by a rat ELISA kit (Shanghai Yi Li Biological Technology Co., Ltd., China).

### Histologic Analyses

To analyze the histology, we fixed tissues with the 10% phosphate-buffered formalin, followed by paraffin embedding, H&E staining, Von Kossa staining, as well as EVG staining by the standard approaches (Wuhan Saville Biotechnology Co., Ltd., China). Imaging of histological sections was performed using a LEICA DM 2000 (Leica Microsystems GmbH, Wetzlar, Germany) microscope. The specific experimental steps are as follows: Fix the rat kidney and blood vessel in 4% paraformaldehyde for more than 24 h, remove the tissue from the fixative solution, trim the target tissue with a scalpel (the kidney and blood vessel are cut longitudinally), and place the cut tissue in the dehydration box Put the gradient alcohol into the dehydrator for dehydration. Put the melted wax into the embedding frame, take the tissue out of the dehydration box and put it into the embedding frame. Cool in a −20° freezer. After the wax solidifies, remove the wax block from the embedding frame and trim the wax block. The wax block was sliced on a paraffin microtome with a thickness of 4 μm. Float the slices on the spreader’s 40°C warm water to flatten the tissues, pick up the tissues with glass slides, and put them in a 60°C oven for baking.

#### H&E Staining

The sections were deparaffinized to water, stained with hematoxylin for 8 min, and rinsed with tap water. Use 1% hydrochloric acid alcohol to differentiate for 30 s, and rinse with tap water 0.6% ammonia water returns to blue, flushing with running water. Finally, tissue sections were stained with eosin for 3 min, and the water in the sections was replaced with a gradient concentration of alcohol. The sections were put into xylene I for 5 min and then xylene II for 5 min. After covered with neutral gum, the sections were observed under a microscope.

#### Von-Kossa Staining

Dewax the section to water, shake off the water on the section, and circle the section with a brush, add Von Kossa dye solution dropwise, irradiate it with a UV lamp for 4 h, and distill to clean it. The sections were stained with hematoxylin staining solution for 5 min and washed with tap water; 1% hydrochloric acid alcohol for 30 s, washed with tap water; 0.6% ammonia water returned to blue, washed with running water. Finally, put in 85% and 95% alcohol gradient dehydration for 5 min, and eosin dye solution for 5 min, and then replace the water in the slice with gradient alcohol. The sections were put into xylene I for 5 min and then xylene II for 5 min. After covered with neutral gum, the sections were observed under a microscope.

#### EVG Staining

The paraffin sections were deparaffinized to water, and the sections were stained with EVG dye solution for 5 min, and rinsed with tap water. Then put the section into EVG dye solution B diluted twice for background differentiation, rinse with tap water, and repeat the operation until the elastic fiber is purple-black and the background is off-white and nearly colorless, and differentiation is over. Re-dye with VG dye solution for 1–3 min, wash in water quickly, and dehydrated quickly in three cylinders with absolute ethanol. Finally, the sections were put into xylene I for 30 s and then xylene II for 5 min. After covered with neutral gum, the sections were observed under a microscope.

### Computed Tomography (CT) Analysis

To evaluate the area of aorta calcification, rats were anesthetized by intraperitoneal injection of 20% urethane and subjected to CT imaging using a PET/CT (SIEMENS Inveon MM, Siemens Ltd., Germany).

### TMT Modified Quantitative Proteomics

The sample was immediately ground to cell powders with liquid nitrogen, followed by transfer to the 5-mL centrifuge tube. Next, 4 volumes lysis buffer consisting of 1% protease inhibitor cocktail and 8 M urea were used to resuspend cell powders under on ice sonication thrice by the use of the high-intensity ultrasonic processor (Scientz). Thereafter, the obtained samples were subjected to 10 min of centrifugation at 12,000 × *g* under 4°C for removing the rest debris. At last, we obtained the supernatants and determined their protein content through the BCA assay in accordance with specific protocols.

Equal amount of protein of each sample was taken for enzymatic hydrolysis, followed by adding an appropriate amount of standard protein and adjusting the volume to the same with lysis buffer. Add one volume of pre-cooled acetone, vortex to mix. Then, 4 volumes pre-chilled acetone were added to precipitate for 2 h under −20°C, followed by 5 min of centrifugation at 4,500 × *g*, later the supernatants were discarded, and pre-chilled acetone was used to rinse the precipitate for two times. After drying the pellet, add TEAB at a final concentration of 200 mM, ultrasonically disperse the pellet. Later, trypsin was added (protease:protein ratio = 1:50, m m^–1^) for overnight hydrolysis. Afterward, dithiothreitol (DTT) was added till the 5 mM final concentration for 30 min reduction under 56°C. Later, iodoacetamide (IAA) was added till the 11 mM final concentration, followed by 15 min incubation in dark under ambient temperature. The TMT kit/iTRAQ kit was used to reconstitute peptide within the 0.5 M TEAB, followed by processing following specific instructions.

The Thermo Betasil C18 column (particle size, 5 μm; length, 250 mm; inner diameter, 4.6 mm) was used for high pH reverse-phase HPLC to fractionate tryptic peptides to different fractions. After dissolving in the mobile phase A of liquid chromatography, we adopted the EASY-nLC 1000 ultra-high performance liquid system to separate the different fractions. The mobile phase A was the aqueous solution consisting of 0.1% formic acid along with 2% acetonitrile, whereas mobile phase B was the aqueous solution consisting of 0.1% formic acid together with 90% acetonitrile. The elution procedure was as follows, 8%∼23% B in 0–38 min; 23%∼35% B in 38–52 min; 35%∼80% B in 52–56 min; and 80% B in 56–60 min, Besides, we fixed the flow rate at 600.00 nL min^–1^. In addition, the ultra-high performance liquid system was used to separate peptides, which were then ionized with the NSI ion source, followed by analysis using the QE + 1 mass spectrometry. The high-resolution Orbitrap was used to detect and analyze peptide precursor ions together with the secondary fragments, at the voltage of ion source of 2.2 kV.

Mass spectroproteomic data has been placed into the Proteome Xchange Consortium through the PRIDE Partner store inventory with the dataset identifier PXD011610. If any additional data is required, it can be obtained from the corresponding author upon reasonable request^1^.

### Mass Spectrometry Quality Control Testing

Many peptides had 7–20 amino acids, which conformed to those general rules related to HCD fragmentation as well as trypsin hydrolysis. Nonetheless, peptides that had <5 amino acids only generated few fragment ions, which were unable to generate the valid sequence identification. Peptides that had over 20 amino acids were not appropriate for HCD fragmentation because of their great charges and mass. Upon mass spectrometry, we found that peptide lengths were distributed in a way conforming to the requirements of quality control (Supplementary Figure 3).

### Quantification of Calcium Deposition

Cells were cultured within the 6-well plates and rinsed by PBS (pH 7.4) thrice, followed by 24 h decalcification using 0.6 mol L^–1^ HCl under 37°C. Afterward, PBS was used to wash cells thrice, followed by 30 min solubilization using the solution consisting of 0.1% SDS and 0.1 mol L^–1^ NaOH. The BCA assay (Beyotime Biotechnology, Cat. #P0012, China) was used to measure protein content. The content of cellular calcium was determined by the QuantiChrom Calcium Assay Kit (Beyotime Biotechnology, Cat. #S1063S, China) and normalized to protein content, which was expressed as μg/mg protein.

### ALP Activity Assay

Cells grown on 6-well plates were washed three times with PBS pH 7.4 and then 150 ul of P0013J (no phosphatase inhibitor) lysis solution was added into each well for 30 min on ice. After lysing cells, we used the alkaline phosphatase (ALP detection kit (Beyotime Biotechnology, Cat. #P0321, China) to determine the ALP activity. Besides, the BCA protein detection kit was utilized to measure protein content. ALP activity was normalized based on protein level and presented in the manner of U/mg protein.

### Alizarin Red Staining

PBS (pH, 7.2–7.4) was used to wash cells, followed by 30 min 4% paraformaldehyde fixation under 37°C. After PBS washing again, cells were subjected to 5 min of 1% Alizarin Red S staining (PH 4.2, Solarbio, Cat. #G1452, China). The Alizarin Red S was discarded and washed 5 times with PBS until the Alizarin Red S was washed away. The culture plate was analyzed under the microscope to capture micrographs. Later, 10% acetic acid was used to counter-stain and dissolve calcium precipitates. For quantifying calcification, we used the multi-detection microplate reader (Dynex, Lincoln, United Kingdom) to measure the OD value at 420 nm.

### Von Kossa Staining

PBS (pH, 7.2–7.4) was used to wash cells, followed by 30 min 4% paraformaldehyde fixation under 37°C. After PBS washing thrice, cells were treated with reagent A of Von Kossa staining Kit (Solarbio, Cat. #G1452, China) and placed under ultraviolet light until calcium phosphate turned black. The dye was washed with PBS three times to remove the excessive dye. After treating with sodium thiosulfate for 2 min, it was washed three times with PBS. The cell nuclei were counterstained with Eosin staining solution (Solarbio, Cat. #G1120). Finally, the culture plate was analyzed under the microscope.

### Western Blot Analysis

The ice-cold cell lysis buffer (Thermo Fisher Scientific) that consisted of the complete phosphatase and protease inhibitor cocktail (Thermo Fisher Scientific) was used to lyse rats’ aortic tissue and VSMCs. Then, the samples were centrifuged for 10 min at 12000 rpm, the Roti-Load1 Buffer (Carl Roth GmbH) was used to boil proteins for 10 min under 100°C. Afterward, we separated equivalent volumes of proteins through SDS-PAGE, followed by transfer onto PVDF membranes. Thereafter, the following primary antibodies were used to incubate the membranes under 4°C overnight: Monoclonal Mouse anti-BMP-2/4 (1:600), Goat anti-Rabbit IgG (H + L) (1:5000), Monoclonal Mouse anti-Runx2 (1:600), Goat anti-Mouse IgG (H + L) (1:5000), Polyclonal Rabbit anti-β-Actin (1:2000), Monoclonal Rabbit anti-TRAF6 (1:5000), Monoclonal Mouse anti-NF-κB p65 (1:500). Later, secondary anti-rabbit IgG (H + L) (1:5000) or anti-mouse IgG (H + L) (1:5000) was used to further incubate the membranes under ambient temperature for 1 h. Finally, we used the stripping buffer (Thermo Fisher Scientific) to strip the membranes for 40 min under ambient temperature for subsequent detection of loading controls. The Odyssey CLX two-color infrared laser imaging system (LI-COR) was employed to obtain protein bands, while ImageJ was used for analysis. All data were presented in the manner of total protein/β-actin ratio or nuclear protein/Lamin A ratio after normalization based on controls.

### Quantitative Real-Time RT-PCR

The RNA Simple Total RNA Kit [Tiangen Biochemical Technology (Beijing) Co., Ltd., Cat. #DP419, China] was used to extract total cellular and tissue RNA following specific protocols. Thereafter, the ReverTra Ace qPCR RT Kit (ToYoBo Life Science, Cat. #FSQ-101, China) was employed to synthesize cDNA from the extracted total RNA through reverse transcription. In addition, the QuantiTect SYBR Green Realtime PCR Master Mix (ToYoBo Life Science, Cat. #QPK-201) was adopted for RT-qPCR by using the Piko Real-Time PCR Detection System (Thermo Fisher Scientific, Cat. #N11471) in accordance with specific protocols. Supplementary Table 1 presents primer sequences.

The specificity was confirmed by analysis of the melting curves of the PCR products. Each PCR procedure was repeated twice, and 2^–Δ^ ^Δ^ ^*Ct*^ method was used to determine the fold changes (FCs) of relative mRNA expression, with GAPDH being the internal control.

### Immunofluorescence

After preparing the slides and performing induction under different conditions, aspirate the supernatant of the 6-well plate, then rinse it with PBS, 1 mL each time, wash twice, and then add 1 mL of 4% paraformaldehyde solution to each well, Fix on ice for 20 min, wash two times with PBS after fixation, add 1 mL of Triton X-100 to each well for cell permeabilization, and repeat washing with PBS three times after 10 min. Add 1 mL of 10% goat serum to each well for blocking for 60 min, wash once with PBS after blocking, pull out the slides from the well plate, add 200 uL of NF-κB (1:800) to each slide, and incubate overnight at 4°C. Collect the primary antibody, wash three times with PBS, add 200 uL of green fluorescent secondary antibody (1:1000) to each slide in the dark, incubate for 1 h in dark (37°C), wash two times with PBS, Add 1 mL of blue fluorescent antibody DAPI to each slide, incubate for 5 min, and wash three times with PBS. After drying in the dark, pull the slide out of the orifice, mount the slide with 1 to 21 drops of anti-fluorescence quencher on the slide, cover the cover glass, and encapsulate the cover glass with nail polish around the cover glass. Capture photos under a confocal microscope.

### Statistical Analysis

Each experiment was carried out for thrice. Data were presented in the manner of mean ± SEM. GraphPad Prism 7.0 was used for statistical analysis and mapping. Multiple group data were analyzed through one-way ANOVA. A difference of *P* < 0.05 indicated statistical significance.

## Results

### Lanthanum Hydroxide Delays the Development of Renal Failure and Protects Renal Function

**FIGURE 2:**
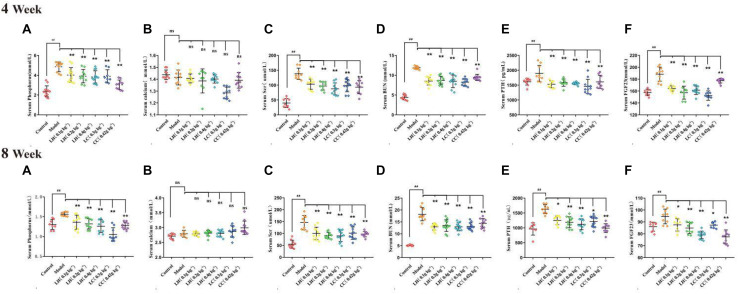
Serum biochemical indicators after lanthanum hydroxide treatment. Serum biochemical examinations for 4 and 8 weeks of drug treatment **(A)** Phosphorus; **(B)** Serum calcium; **(C)** Scr, serum creatinine; **(D)** BUN, serum urea nitrogen; **(E)** PTH, parathyroid hormone; **(F)** FGF23, fibroblast growth factor 23 (mean ± SEM, *^##^P* < 0.01 compared with the control group; *P < 0.05, **P < 0.01 compared with the model, NS, not significant, *N* = 9–11).

The CKD rat modeling method is shown in Figure 1. In order to evaluate the protective effect of lanthanum hydroxide on the kidneys of CKD rats, we measured the levels of serum phosphorus, calcium, creatinine, and urea nitrogen at 4 and 8 weeks after treatment. According to Figures 2, 4 week compared with the control group, the serum phosphorus, creatinine, and urea nitrogen of the model group increased by 4.85 ± 0.50, 0.31 ± 0.05, 11.88 ± 0.38, respectively, and compared with the normal control group, there were significant differences (*P* < 0.01), suggesting that adenine combined with a 1.2% high-phosphorus diet was successfully modeled. After 4 weeks of administration of lanthanum hydroxide, compared with the model group, serum phosphorus, creatinine, and urea nitrogen levels were significantly reduced. As shown in Figure 2, after 8 weeks of treatment, it can be seen that the lanthanum hydroxide 0.4 g kg^–1^ group has the best phosphorus-lowering effect, and the levels of blood urea nitrogen (BUN) and serum creatinine (Scr) decreased depending on its dose. There was no statistical difference in the detection results of serum calcium at 4 and 8 weeks after administration.

**FIGURE 3:**
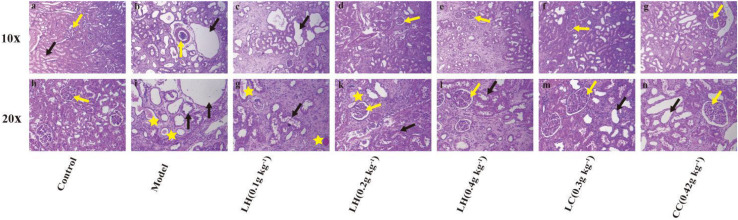
Representative histopathological findings in kidney tissues. As shown in the figure, compared with the control group, the kidney pathology of the model group is: glomerular necrosis and disappearance (yellow arrow), renal tubule dilation (black arrow), protein cast (yellow star), and inflammatory factors Infiltration, and fibrosis of the renal interstitium appears. After treatment with lanthanum hydroxide, the glomerular structure was relatively complete, the degree of renal tubule dilatation was significantly improved, and the inflammatory infiltration was also reduced. **(a–g)** Kidney pathology of each group of animals at 100 times. **(h–n)** Kidney pathology of each group of animals at 200 times.

According to the results of HE staining of rat kidney in Figure 3, compared with the control group, the degeneration and necrosis of the proximal convoluted tubule epithelial cells in the renal cortex and the disappearance of the nuclei can be observed in the other groups. The mesenchyme is accompanied by a large number of mononuclear cell infiltration, glomerular necrosis and disappearance, and visible protein casts and obvious expansion of the renal tubules. The model group exhibited obvious pathological changes, including chronic granulomatous inflammation and purine deposits in some renal tubules (Figures 3B,I). At the same time, there were also white blood cell casts and renal mesenchymal fibrous tissue focal hyperplastic lesions in the renal tubules. The pathological results of the lanthanum hydroxide (0.4 g kg^–1^, 0.2 g kg^–1^, and 0.1 g kg^–1^) group showed that compared with the model group, cell deformation and infiltration were lighter, and the degree of renal tubule expansion was significantly improved. Renal interstitial hyperplasia is also effectively controlled, and the glomerular structure is relatively complete (Figures 3C–E,G,K,L). It was observed that, lanthanum hydroxide can significantly reduce serum phosphorus levels, protect the kidneys, and slow the development of renal failure.

### Lanthanum Hydroxide Inhibits the Development of Vascular Calcification

The above results show that lanthanum hydroxide can effectively reduce serum phosphorus levels and defer the progression of renal failure. Because the increase in blood phosphorus is closely related to vascular calcification, we measured serum PTH, and FGF23 levels, and performed computed tomography (CT) examination of rat thoracic aorta and Von Kossa staining of tissue sections.

Relative to the control group, the CKD model group exhibited markedly elevated serum of levels FGF23 and PTH. After treatment with lanthanum hydroxide, the serum PTH and FGF23 levels decreased, and the difference was significant relative to model group (*P* < 0.01) (Figures 2E,F). Elevated blood phosphorus can lead to increased levels of PTH and FGF23, the latter being a major regulator of serum phosphorus synthesized and released by osteoblasts, which plays a role in the metabolism of vitamin D and secondary hyperparathyroidism. It also plays a key role and is directly involved in vascular calcification. Lanthanum hydroxide can slow the occurrence of vascular calcification by reducing serum phosphorus, thereby reducing the levels of PTH and FGF23 in the serum.

**FIGURE 4:**
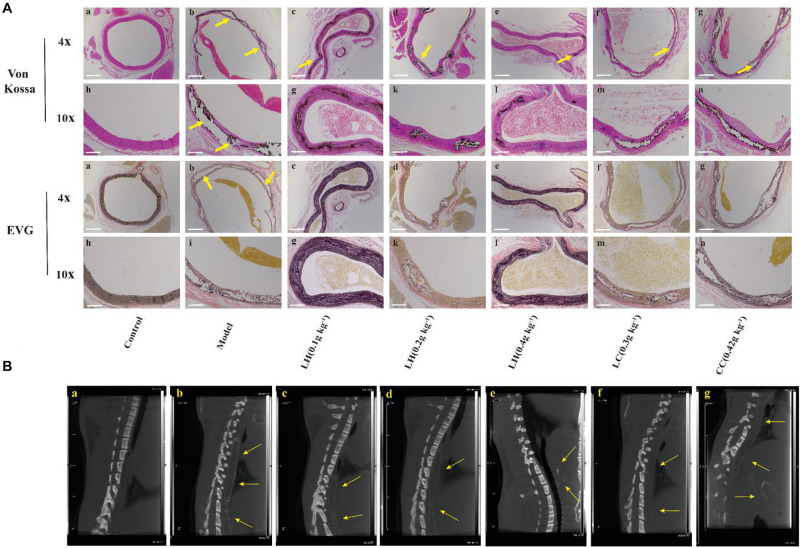
Representative computed tomography (CT) images of ectopic calcification and micrographs of Von Kossa and EVG stained sections of the thoracic aorta after 12 weeks. **(A)** Von Kossa and EVG stained tissue image of thoracic aorta. **(B)** Ectopic calcification was observed in thoracic aortic sections on CT in model group (yellow arrows). **(a)**, control group; **(b)**, model group; **(c)**, lanthanum hydroxide (0.1 g kg^–1^); **(d)**, lanthanum hydroxide (0.2 g kg^–1^); **(e)**, lanthanum hydroxide (0.4 g kg^–1^); **(f)**, lanthanum carbonate (0.3 g kg^–1^); **(g)**, calcium carbonate (0.42 g kg^–1^). Von Kossa and EVG staining positive legions were observed in CKD rat (yellow arrows). Scale bar, 200 μm.

Figure 4 shows the EVG and Von Kossa staining of thoracic aortic slices. Vascular calcification was not observed in aortic sections of controls (Figures 4Aa,h). In contrast, in the model groups, there was obvious evidence (positive Von Kossa) of vascular calcification exclusively in the media. It is noteworthy that the elastin layers in these calcified areas appeared to be disorganized and disrupted in a gross manner (Figures 4Ab,i). After 0.4 g kg^–1^ lanthanum hydroxide treatment, a significant reduction in calcification was observed (Figures 4Ae,l), as well as less elastic fiber breakage has also been improved. There was obvious aortic calcification in the model group on CT images (Figure 4Ba, yellow arrows), and the aortic calcification decreased in the 0.4% lanthanum hydroxide group (Figure 4Bb).

### The Proteomic Analysis of Aortic Tissue After Lanthanum Hydroxide Treatment *in vivo*

In order to further explore how lanthanum hydroxide delays the occurrence of vascular calcification, we analyzed the effect of lanthanum hydroxide on the CKD rat model through quantitative proteomics technology, and investigated the effect of expression changes due to lanthanum hydroxide on differential proteins in order to verify the relevant signal pathways and provide evidence.

**FIGURE 5:**
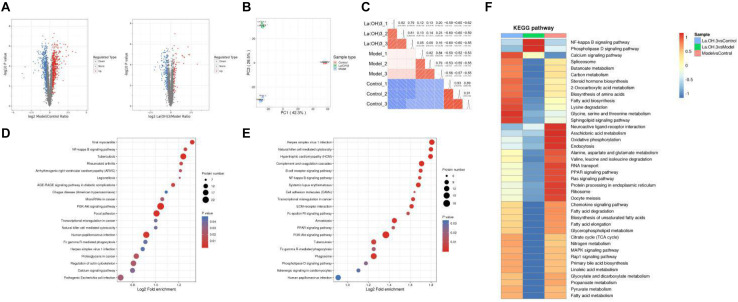
Proteomic analysis of aortic tissues. **(A)** Differential protein quantitative volcano map. The horizontal axis in the figure stands for relative protein level after Log2 conversion, while the vertical axis represents *P*-value obtained upon significance test following -Log10 conversion. In the figure, the red and blue dots stand for significantly upregulated and downregulated proteins, respectively. **(B)** The 2D scatter plot displaying PCA for protein quantification across duplicated samples. **(C)** Pearson’s correlation coefficient heat map. **(D)** KEGG pathway enrichment analysis-Model vs. Control. **(E)** KEGG pathway enrichment analysis-La(OH)_3_ (0.4 g kg^–1^) vs. Model. **(F)** Cluster analysis heat map of KEGG pathway. The similar functions from diverse groups are clustered and plotted in the heat map based on *P*-values upon enrichment test by the hierarchical clustering approach, where horizontal axis stands for diverse comparison groups, while vertical axis represents functional description of DEPs. The diverse color blocks stand for enrichment levels, where red and blue stand for strong and weak enrichment, respectively.

To study the differences in aortic proteomics between the control group and the CKD rat model group, tandem mass tag (TMT) labeling, mass spectrometry-based quantitative proteomics, and high-performance liquid chromatography (HPLC) fractionation technologies were applied. Significantly upregulated proteins were selected at the thresholds of FC > 1.3 and *P* < 0.05, whereas significantly downregulated proteins were selected according to FC < 1/1.3. The differential protein quantitative volcano chart is shown in Figure 5A. On the horizontal axis in the figure, the relative protein expression is converted into log2 value, while the *P*-value obtained from the significance test after being converted to −log10 are on the vertical axis. In the figure, the red and blue dots denote significantly upregulated and downregulated proteins, respectively. Clearly, relative to the control group, there were 448 significantly upregulated and 406 downregulated proteins identified in the CKD model group. Relative to the CKD model group, 69 proteins were significantly upregulated after lanthanum hydroxide administration, and 168 proteins were significantly downregulated.

For biological or technical replicate samples, it is necessary to test the statistical consistency between the results obtained for biological samples and technical duplicate samples. We conducted two analysis methods, principal component analysis (PCA) and Pearson correlation, to evaluate the repeatability of protein quantification. PCA analysis shows that the control, model, and lanthanum hydroxide (0.4 g kg^–1^) groups have good repeatability and can be completely separated (Figure 5B). Pearson correlation analysis also shows that the data has a satisfactory linear correlation (Figure 5C). The CKD rat model induced by adenine combined with a 1.2% high-phosphorus diet was successful, and the treatment of lanthanum hydroxide also had a certain effect on the model rats.

For clarifying the enrichment trend of differentially expressed proteins (DEPs) in some functions, the Kyoto Encyclopedia of Genes and Genomes (KEGG) pathway analysis was performed based on all the screened DEPs. A bubble chart was plotted to present the significantly enriched DEPs based on the *p*-values obtained from the enrichment test (*P* < 0.05). The vertical axis in Figures 5D,E represents the functional classification or pathway, whereas the horizontal axis denotes the log2-transformed value regarding the proportion of DEPs in a function type to all proteins identified. The circle color indicates the *P*-value upon enrichment significance test, while the circle size represents the number of DEPs involved in the pathway. According to the KEGG enrichment analysis of the control, model, and lanthanum hydroxide (0.4 g kg^–1^) groups, they can be enriched into the nuclear factor-κB (NF-κB) pathway, PI3K-AKT signaling pathways and cardiovascular disease-related pathways.

To determine the correlation of the differentially modified protein functions after lanthanum hydroxide treatment, we performed KEGG cluster analysis when DEPs were enriched to diverse groups (Figure 5F). We clustered similar functions from diverse comparison groups to draw a heat map based on *p*-values acquired upon enrichment test by hierarchical clustering, where the horizontal axis indicated diverse comparison groups, and the vertical axis describes the corresponding differentially expressed gene (DEG) functions. The diverse color blocks indicate the enrichment level, and red and blue denote strong and weak enrichment, respectively. Relative to the model group, DEPs were mostly associated with the NF-κB signal transduction pathway following treatment with lanthanum hydroxide.

The high CVD morbidity and mortality among CKD cases are primarily ascribed to arterial media vascular calcification. Therefore, controlling the occurrence and development of vascular calcification is the key to a positive prognosis for CKD patients. Vascular calcification has been identified as an active process under the regulation of VSMCs. VSMCs can change their phenotype and release stromal vesicles under pathological conditions, which in turn trigger the occurrence of aortic calcification. The signaling pathway regulating the phenotypic transdifferentiation of VSMCs is complex, and it involves systemic inflammation and at least partly depends on the transcription factor NF-κB. It has been demonstrated that NF-κB is a key regulator of vascular calcification. Voelkl et al. (2018b) showed that zinc can inhibit the activation of the NF-κB signal pathway through GPR39-dependent TNFAIP3, thereby inhibiting phosphate-induced osteoblast-like cell transdifferentiation of VSMCs and slowing the development of vascular calcification.

Through a summary of the relevant literature and proteomic analysis of aortic tissue, the NF-κB signal transduction pathway was utilized to investigate how lanthanum hydroxide affects hyperphosphatemia-mediated vascular calcification in the context of CRF.

### Lanthanum Hydroxide Inhibits Phosphate-Induced Calcification of VSMCs Through Suppression of NF-κB *in vitro*

**FIGURE 6:**
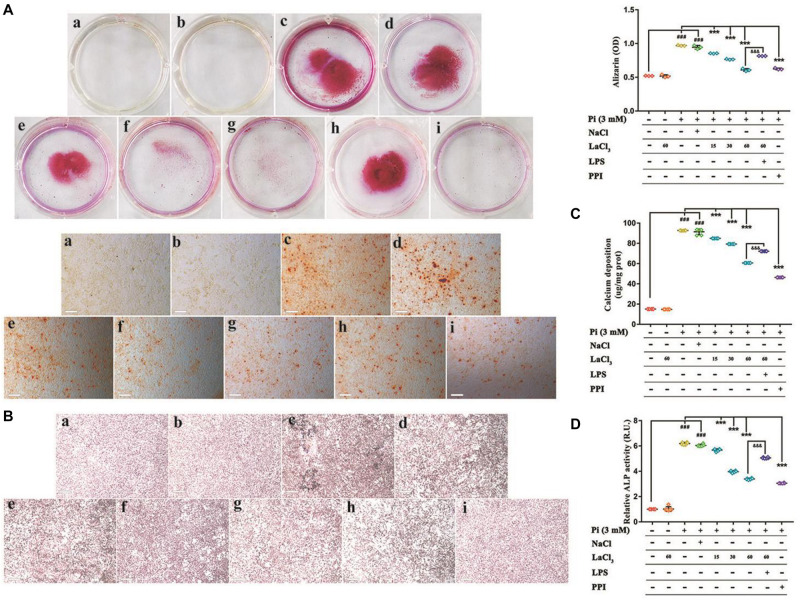
Lanthanum suppresses the phosphate-mediated vascular mineralization depending on its dose by suppressing NF-κB. VSMCs were grown for 6 days within the calcification medium that contained 3 mmol/L phosphate (Pi) with or without 15–60 μM lanthanum **(A–D)**. **(A)** Alizarin Red staining was conducted to visualize calcium deposits. Typical wells as well as images for the stained plates upon 3 individual experiments. Quantification of Alizarin Red to represent the amount of calcium (*n* = 3). **(a)**, Control group (1 mM phosphate buffer); **(b)**, Control + LaCl_3_ (60 μM) group; **(c)**, Model group (3 mM phosphate buffer); **(d)**, NaCl group (180 μM); **(e)**, LaCl_3_ low dose group (15 μM); **(f)**, LaCl_3_ medium dose group (30 μM); **(g)**, LaCl_3_ high dose group (60 μM); **(h)**, LPS group (1 ug mL^–1^); **(i)**, PPI group (100 μM). **(B)** Calcium deposition was visualized by Von Kossa Staining. Typical wells as well as images for the stained plates upon three individual experiments. **(a)**, Control group (1 mM phosphate buffer); **(b)**, Control + LaCl_3_ (60 μM) group; **(c)**, Model group (3 mM phosphate buffer); **(d)**, NaCl group (180 μM); **(e)**, LaCl_3_ low dose group (15 μM); **(f)**, LaCl_3_ medium dose group (30 μM); **(g)**, LaCl_3_ high dose group (60 μM); **(h)**, LPS group (1 ug mL^–1^); **(i)**, PPI group (100 μM). **(C)** Scatter plots along with arithmetic means ± SEM (*n* = 6, ug/mg protein) for calcium deposits within VSMCs following 6 days of treatment with Pi or control, with or without 15–60 μM LaCl_3_. **(D)** Scatter plots together with arithmetic means ± SEM (*n* = 6, U/mg protein) for the ALP activity within VSMCs following 6 days of treatment with Pi or control, with or without 15–60 μM LaCl_3_. All values are presented in the manner of mean ± SEM from four individual experiments. One-way ANOVA test was adopted for statistical analysis. The significance level was set at *p* < 0.05; ^###^*P* < 0.001 versus control VSMCs; ***P < 0.001 versus Pi-treated VSMCs. ^&⁣&⁣&^*P* < 0.001 versus Pi + LaCl_3_ (60 μM)-treated VSMCs. Scale bar = 200 μm.

To determine whether the promotion of calcification was due to reduced cell viability, the effect of LaCl_3_ on cytotoxicity was investigated by MTT assay. As shown in Supplementary Figure 1, it was determined that the low, medium and high doses for LaCl_3_ were 15, 30, and 60 μM, respectively, and the incubation time was 48 h. LaCl_3_ has been shown to treat chronic renal failure and hyperphosphatemia. To examine the inhibitory effect of LaCl_3_ on Pi-stimulated VSMCs mineralization via suppressing NF-κB under the conditions in this experiment, we grew VSMCs in calcification medium that contained 3 mM inorganic Pi with or without 15–60 μM LaCl_3_ as well as with or without 1 ug mL^–1^ lipopolysaccharides (LPS) for 6 days. We performed Von Kossa and Alizarin Red Staining to detect granular deposits. Relative to untreated cells, Pi caused a significant 2-fold increase in mineral deposition, while the exposure of VSMCs to LaCl_3_ decreased Pi-induced mineralization in a dose-dependent manner. Importantly, 60 μM LaCl_3_ decreased mineral deposition in VSMCs exposed to high Pi, so that it was at the level of cells maintained in growth medium (Figures 6A,B). The exposure of VSMCs to LaCl_3_ resulted in a 2/3 decrease in calcium deposition and alkaline phosphatase (ALP) activity (Figures 6C,D). After adding LPS, compared with the high-dose LaCl_3_ group, the calcium deposition and ALP activity of the LPS group significantly increased, and the calcium deposition almost reached that for the mid-dose level of LaCl_3_. In addition, differences in ALP activity and calcium level between the 60 μM LaCl_3_ group (without inorganic Pi) and the control group or between the model group and the NaCl group were not significant, indicating that Cl^–^ had no effect on calcification, and lanthanum was effective. The above findings suggest that LaCl_3_ efficiently suppressed Pi-mediated mineral deposition by suppressing NF-κB within human VSMCs in a dose-dependent manner.

### Effects of Lanthanum Hydroxide Supplementation on Vascular Calcification During Chronic Renal Failure *in vivo* and *in vitro*

**FIGURE 7:**
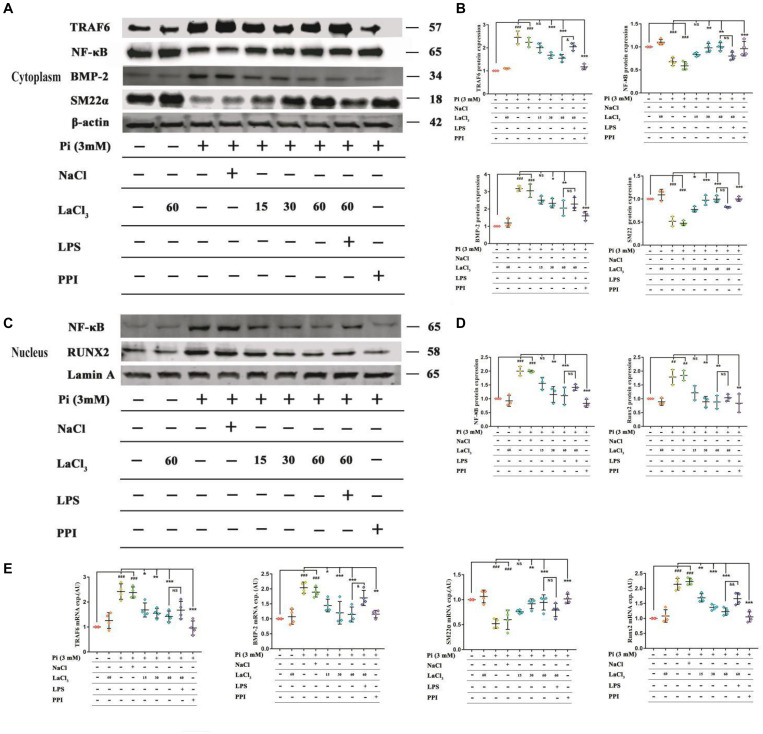
LaCl_3_ application mitigates vascular calcification and suppresses the osteoinduction signal transduction in the context of CRF. **(A)** Typical Scatter plots, Western blots as well as arithmetic means ± SEM (*n* = 3) of normalized cytoplasmic TRAF6/β-actin, NF-κB/β-actin, BMP-2/β-actin, and SM22α/β-actin protein ratio in VSMCs with control or with high phosphorus without or with additional treatment with LaCl_3_. **(B)** Quantification of Western blots to represent the amount of cytoplasmic protein expression (*n* = 3). **(C)** Typical Scatter plots, Western blots as well as arithmetic means ± SEM (*n* = 3) for the standardized nuclear NF-κB/Lamin A and Runx2/Lamin A protein ratio in VSMCs with control or with high phosphorus without or with additional treatment with LaCl_3_. **(D)** Quantification of Western blots to represent the amount of nuclear protein expression (*n* = 3). **(E)** Scatter plots and arithmetic means ± SEM (*n* = 3) of TRAF6, BMP-2, SM22α, and Runx2, relative mRNA expression in VSMCs with Pi or control, with or without LaCl_3_ treatment. ^##^*P* < 0.01; ^###^*P* < 0.001 compared with control VSMCs; *P < 0.05; **P < 0.01; ***P < 0.001 compared with Pi-treated VSMCs. ^&^*P* < 0.05; ^&⁣&^*P* < 0.01 compared with VSMCs treated with Pi and LaCl_3_ (60 μM).

**FIGURE 8:**
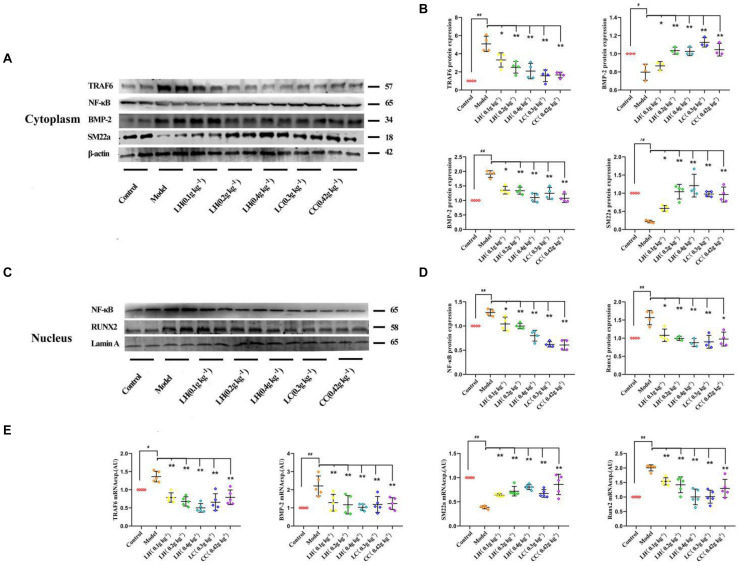
Lanthanum hydroxide supplementation ameliorates vascular calcification and osteoinductive signaling in chronic renal failure. **(A)** Typical Scatter plots, Western blots as well as arithmetic means ± SEM (*n* = 3) of the standardized cytoplasmic TRAF6/β-actin, NF-κB/β-actin, BMP-2/β-actin, and SM22α/β-actin protein ratio in rats with Pi or control, with or without lanthanum hydroxide treatment. **(B)** Quantification of Western blots to represent the amount of cytoplasmic protein expression (*n* = 3). **(C)** Typical Scatter plots, Western blots as well as arithmetic means ± SEM (*n* = 3) for standardized nuclear NF-κB/Lamin A and Runx2/Lamin A protein ratio in rats with control or with high phosphorus without or with additional treatment with lanthanum hydroxide. **(D)** Quantification of Western blots to represent the amount of nuclear protein expression (*n* = 3). **(E)** Scatter plots and arithmetic means ± SEM (*n* = 3) of TRAF6, BMP-2, SM22α, and Runx2, relative mRNA expression in rats with Pi or control, with or without lanthanum hydroxide treatment. ^#^*P* < 0.05; ^##^*P* < 0.01 compared with control rats; *P < 0.05; **P < 0.01 compared with model rats.

To explore the role of lanthanum hydroxide in inhibiting calcification, we initially turned to a robust cell-culture-based −3 mM inorganic Pi-induced Western blot and real-time PCR assay in the VSMCs. The results showed that compared with the control group, after high Pi induced VSMCs, the expression of smooth muscle SM (SM22α) at gene and protein levels was significantly downregulated, and the osteogenic markers runt-related transcription factor 2 (Runx2) and bone morphogenetic protein 2 (BMP-2) markedly increased (Figures 7A–E). Therefore, it can be proved that VSMCs underwent osteogenic transdifferentiation. In order to verify whether lanthanum chloride regulates the occurrence of vascular calcification through the NF-κB pathway, we measured NF-κB and TNF receptor associated factor 6 (TRAF6) protein levels. We found that there were markedly elevated nuclear NF-κB and TRAF6 levels in the model group relative to those in control group, while their expression decreased in a concentration-dependent fashion when exposed to LaCl_3_. After using the NF-κB agonist LPS, compared with LaCl_3_ (60 μM), there was an increasing trend, but it was not significant (Figures 7A–D). Because LaCl_3_ inhibits the activation of the NF-κB pathway, the expression of SM22α is increased, and the expression of Runx2 and BMP-2 is decreased, thereby preventing the occurrence of VSMCs mineralization. At the same time, we also performed Western blot and realtime PCR detection of the aorta of CKD rats. The expression of TRAF6, nuclear NF-κB, BMP-2, and Runx2 was increased in the established CRF model. After 8 weeks of continuous lanthanum hydroxide treatment, the protein levels of TRAF6, nuclear NF-κB, BMP-2, and Runx2 decreased in a dose-dependent manner in each group (Figures 8A–E). In addition, we detected nuclear translocation of NF-κB by immunofluorescence. As shown in Supplementary Figure 2, under high phosphorus conditions, nuclear translocation of NF-κB occurred, and after administration of LaCl_3_ (60 μM), the amount of NF-κB in the nucleus was significantly reduced. The results are basically consistent with the results of *in vitro* studies.

It was observed that lanthanum hydroxide may inhibit the activation of NF-κB, reduce the expression of osteogenic markers, and thereby inhibit the occurrence of calcification.

## Discussion

Lanthanum hydroxide, a new phosphate binder, delayed the progression of kidney failure, suppressed the development of vascular calcification; decreased serum PTH and FGF23 levels, and significantly suppressed serum phosphorus. Thus, lanthanum hydroxide inhibited the progression of vascular calcification by decreasing serum phosphorus levels.

Diverse animal models are utilized for investigating the CKD-related MAC formation. Adenine has become an important xanthine dehydrogenase substrate, which can be oxidized to 2,8-dihydroxyadenine. Due to its low solubility, 2,8-dihydroxyadenine can be deposited within renal tubules in the form of stones, giving rise to nephrolithiasis, fibrosis, necrosis, wide tubular dilation, and renal dysfunction (Tamagaki et al., 2006; Tamura et al., 2009). The adenine model has the advantage of inducing renal failure over a short period of time with very little variations between animals (Tamagaki et al., 2006; Massy et al., 2007). We induced a CKD model by combining adenine with a high-phosphorus diet (1.2% -phosphorus), and the weight loss and increases in creatinine and BUN observed in the CKD rats were similar to findings described in previous reports. The findings in this work conformed to those obtained by Jia et al., who discovered that an adenine-supplemented diet increased FGF-23 and PTH as the administration time prolonged (Jia et al., 2013). In the later stages of CKD in animal models, bone abnormalities and vascular calcification were observed. According to human studies, aberrant biochemical changes were detected at the GFR was 40–50 ml min^–1^ (CKD 3–4); meanwhile, bone diseases and vascular calcification lesions were commonly seen among CKD stage 4-5D patients (Block et al., 2005; Mehrotra and Adler, 2005; Levin et al., 2007; Russo et al., 2007). This model displays the characteristics of progressive onset and the course of chronic renal failure.

In the current study, lanthanum hydroxide treatment provided a satisfactory therapeutic effect in a rat model of hyperphosphatemia renal failure. By measuring physiological and biochemical indicators, the level of serum phosphorus was decreased compared with the model group, and the phosphorus-lowering effect was reduced in a dose-dependent manner after treatment with lanthanum hydroxide. Treatment with 0.2 g kg^–1^ lanthanum hydroxide decreased serum phosphorus, PTH, and FGF23 levels, and delayed the development of renal failure. The same improvements were observed after 0.3 g kg^–1^ lanthanum carbonate and 0.42 g kg^–1^ calcium carbonate treatments were administered. These results indicated that the effect of lanthanum hydroxide was similar to that of existing drugs. However, 0.4 g kg^–1^ lanthanum hydroxide achieved more optimal therapeutic effects when compared to the effects produced by lanthanum carbonate and calcium carbonate, With the use of our renal failure model using hyperphosphatemia rats, the therapeutic advantage of lanthanum hydroxide was determined.

Fibroblast growth factor 23 is secreted by bone cells and osteoblasts, and its main target organs are the kidneys and parathyroid glands. It is a recently discovered important factor that regulates calcium and phosphorus metabolism. Mirza et al. (2009) found that higher or normal serum levels of FGF23 were independently related to the aggravated vasoreactivity impairment and arterial stiffness. CKD patients with high FGF-23 are more likely to have impaired vascular reactivity as well as vascular calcification as compared to patients with a normal glomerular filtration rate. Desjardins et al. (2012) demonstrated that increased FGF-23 levels were positively correlated with the arterial calcification index. Relative to the control group, the model group exhibited elevated FGF23 (94.55 mmol mL^–1^), and after treatment with 0.4 g kg^–1^ lanthanum hydroxide after 8 weeks, the serum FGF23 decreased to 79.10 mmol mL^–1^ (Figures 2F, 8 week). The Von Kossa staining and CT imaging of the thoracic aorta in rats after 8 weeks of treatment showed that the calcification in the 0.4 g kg^–1^ lanthanum hydroxide group markedly decreased relative to that in the model group (Figure 4).

NF-κB is a key regulator of vascular calcification. Under *in vitro* conditions, high phosphorus can promote nuclear translocation of NF-κB. The overexpression of glucocorticoid-induced kinase 1 (SGK1) promotes the transdifferentiation and calcification of VSMCs into osteoblasts, and the above process depends on the activation of NF-κB. Under *in vivo* conditions, after knocking out SGK1, NF-κB activity was significantly reduced and calcification was significantly weakened (Voelkl et al., 2018a). Under high phosphorus conditions, the mineral metabolism of mice is disturbed. Growth hormone releasing hormone agonist (GHRH-A) inhibits the production of Runx2 and ALP by inhibiting the activity of NF-κB, thereby inhibiting the transdifferentiation of VSMCs. After injection of GHRH-A, the vascular calcification of mice was also significantly weakened. Therefore, inflammation-mediated VSMCs transdifferentiation into osteoblasts and vascular calcification are blocked (Shen et al., 2018). As for the cultured VSMCs, LaCl_3_ exposure mitigated the phosphate-mediated mineral precipitation. We conducted Alizarin Red and Von Kossa staining to detect granular deposits. Compared with the untreated cells, Pi caused a significant 2-fold increase in mineral deposition, whereas exposure of VSMCs to LaCl_3_ reduced the Pi-mitigated mineralization in a dose-dependent manner. Importantly, 60 μM LaCl_3_ decreased mineral deposition in VSMCs treated with high levels of Pi to that maintained within the growth medium (Figure 6). Therefore, both *in vitro* and *in vivo* studies have shown that lanthanum hydroxide may inhibit the occurrence and development of vascular calcification. The mechanisms underlying the vascular calcification occurrence and development are similar to those related to physiological osteogenesis, which involve vascular cell apoptosis, osteo-/chondrogenic transdifferentiation, reduced calcification inhibitor, extracellular matrix (ECM) remodeling, and release of extracellular vesicles (Shanahan et al., 2011; Alves et al., 2014; Schlieper et al., 2016). These mechanisms are not mutually exclusive. VSMCs play a key role during vascular calcification (Desjardins et al., 2012; Lanzer et al., 2014; Voelkl et al., 2019). VSMCs can change to the osteo-/chondroblast-like cell phenotype, so as to aggressively respond to increased extracellular phosphate and enhance vascular mineralization (Alesutan et al., 2017; Luong et al., 2018; Voelkl et al., 2019). The transdifferentiated VSMCs have lost the contractile phenotype to develop the mesenchymal phenotype and to obtain characteristics similar to those of chondroblasts or osteoblasts (Demer and Tintut, 2008). In such VSMCs, smooth muscle-specific protein expression decreases, such as smooth muscle SM22α and α-smooth muscle actin (αSMA) (Steitz et al., 2001), whereas osteogenic transcription factor levels are upregulated, like homeobox 2 (MSX2) and core-binding factor α-1 (CBFA1). Among them, CBFA1 (also referred to as Runx2) plays an important role in vascular calcification. Additionally, these osteo-/chondrogenic transcription factors are used to further induce chondrogenic or osteogenic protein expression within VSMCs, such as that which will produce type I collagen, osteocalcin, ALP, and BMP-2 (Lanzer et al., 2014; Leibrock et al., 2016; Voelkl et al., 2019). Therefore, these transdifferentiated VSMCs potentially enhance vascular calcification through generating a local environment that promotes calcification, together with the nidus sites for phosphate/calcium precipitation and calcium phosphate crystal growth. However, additional studies are required to understand the complicated events resulting in vascular calcification within CKD.

**FIGURE 9:**
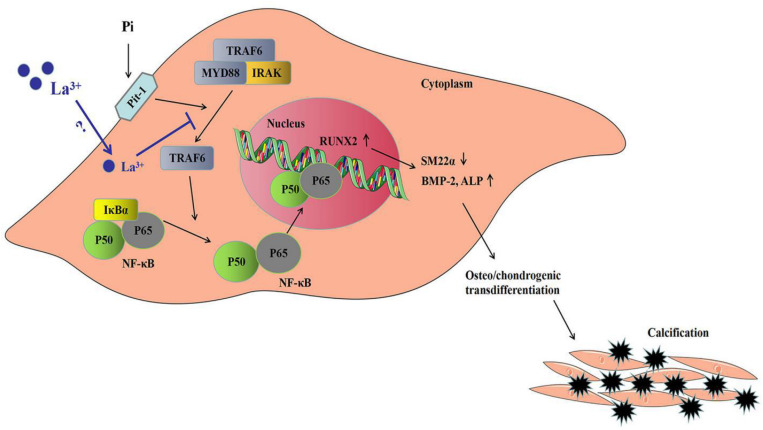
Sketch map regarding the phosphate-mediated VSMC calcification and the suppression by zinc application. Treatment with great phosphate (Pi) content triggers the activation of NF-κB (p50/p65) within VSMCs. The activity of NF-κB is determined by IκBα degradation and ubiquitination according to phosphorylation. The active transcription factor can translocate to the nucleus and induce the target gene expression for promoting VSMCs osteo-/chondrogenic transdifferentiation. Such transdifferentiation can generate the pro-calcifying environment to induce vascular mineralization. Lanthanum supplementation inhibits TRAF6 protein levels. The suppressed TRAF6 protein expression inhibits the activation of NF-κB, thus suppressing VSMCs osteo-/chondrogenic transdifferentiation, decreasing the formation of the pro-calcifying environment as well as the later mineralization.

In this study, to analyze the mechanisms of lanthanum hydroxide in delaying vascular calcification, Western blotting was performed using the proteins of the thoracic aorta and VSMCs of rats. The results showed that compared with the control group, SM22α decreased and BMP-2 significantly increased in the model group, indicating that VSMCs were transformed into osteoblasts. Subsequently, we identified NF-κB as a key regulator of VSMCs phenotype and calcification. We demonstrated that lanthanum supplementation provides a remarkable protective effect during vascular calcification *in vitro* and in animal models. A high phosphate content may lead to an increased risk of vascular calcification, while the application of lanthanum mitigates osteo-/chondrogenic transdifferentiation of VSMCs. Lanthanum inhibits TRAF6 expression, and later suppresses NF-κB activation or osteo-/chondrogenic reprogramming within VSMCs in the process of hyperphosphatemia. However, the direct inhibitory mechanism of La^3+^ on the NF-κB signaling pathway is not very clear. Therefore, which type of receptor La^3+^ acts on to inhibit vascular calcification needs further study (Figure 9).

## Conclusion

Our study demonstrated that lanthanum hydroxide, a new type of phosphorus binder, can effectively reduce serum phosphorus levels, delay the process of renal failure, and protect renal function. Moreover, lanthanum hydroxide may delay the occurrence and development of vascular calcification through suppressing NF-κB pathway activation via La^3+^. Therefore, our foundational data can be applied to pre-clinical pharmacological research so that lanthanum hydroxide may become a clinical candidate drug.

## Data Availability Statement

The datasets presented in this study can be found in online repositories. The names of the repository/repositories and accession number(s) can be found below: PRIDE database, PXD023081.

## Ethics Statement

The animal study was reviewed and approved by the Inner Mongolian Medical University.

## Author Contributions

LZ and SW performed the experiments, analyzed the data, and drafted the manuscript. HL, XD, and RB assisted with the experiments. BL, RH, JG, YL, JH, JZ, and YM provided suggestions to the study design and writing of the manuscript. GL designed and financed the study, and wrote and edited the manuscript. All authors contributed to the article and approved the submitted version.

## Conflict of Interest

The authors declare that the research was conducted in the absence of any commercial or financial relationships that could be construed as a potential conflict of interest.

## Footnotes

1 http://www.ebi.ac.uk/pride
