# Supplementary material for: The Pharmacological Effect and Mechanism of Lanthanum Hydroxide on Vascular Calcification Caused by Chronic Renal Failure Hyperphosphatemia
Source: Front Cell Dev Biol. 2021 Apr 13;9:639127. doi: 10.3389/fcell.2021.639127 (PMC8076751; doi:10.3389/fcell.2021.639127)
Supplement: Supplementary file 1 [file Data_Sheet_1.pdf]

Supplementary Material

1 Supplementary Figures and Tables

1.1 Supplementary Figures

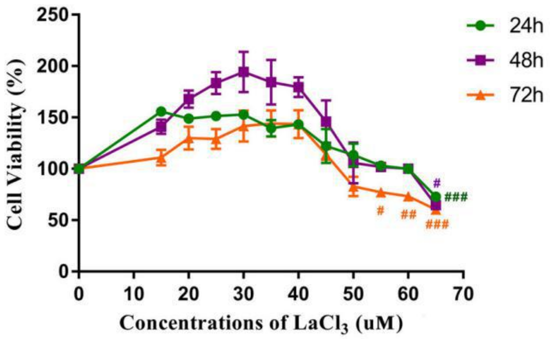

Supplementary Figure 1. The cell viability was detected by the MTT method to determine the incubation concentration. VSMCs were cultured for 24h, 48h and 72h cell viability under high phosphorus conditions. All values are presented in the manner of mean ± SEM from 4 individual experiments. One-way ANOVA test was adopted for statistical analysis. The significance level was set at $p < 0.05$. $^{\#}P < 0.05$; $^{\#\#}P < 0.01$; $^{\#\#\#}P < 0.001$ versus $LaCl_3$ (0 uM).

1.2 Supplementary Figures

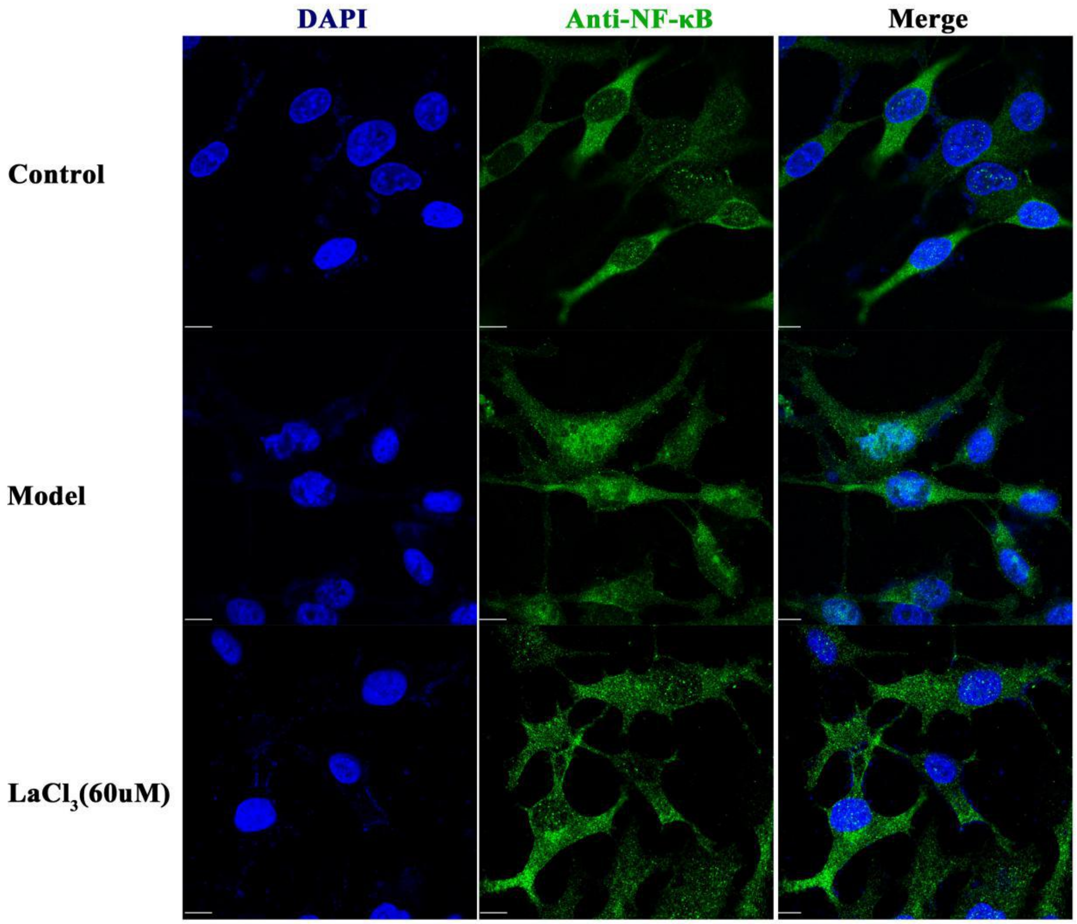

Supplementary Figure 2. Role of NF-κB in High phosphorus-induced osteo-/chondrogenic transdifferentiation of VSMCs. Microscopy images of control, Model and $LaCl_3$ primary cultured VSMCs stained with anti-NF-κB antibodies and DAPI. Scale bar =10 μm

1.3 Supplementary Figures

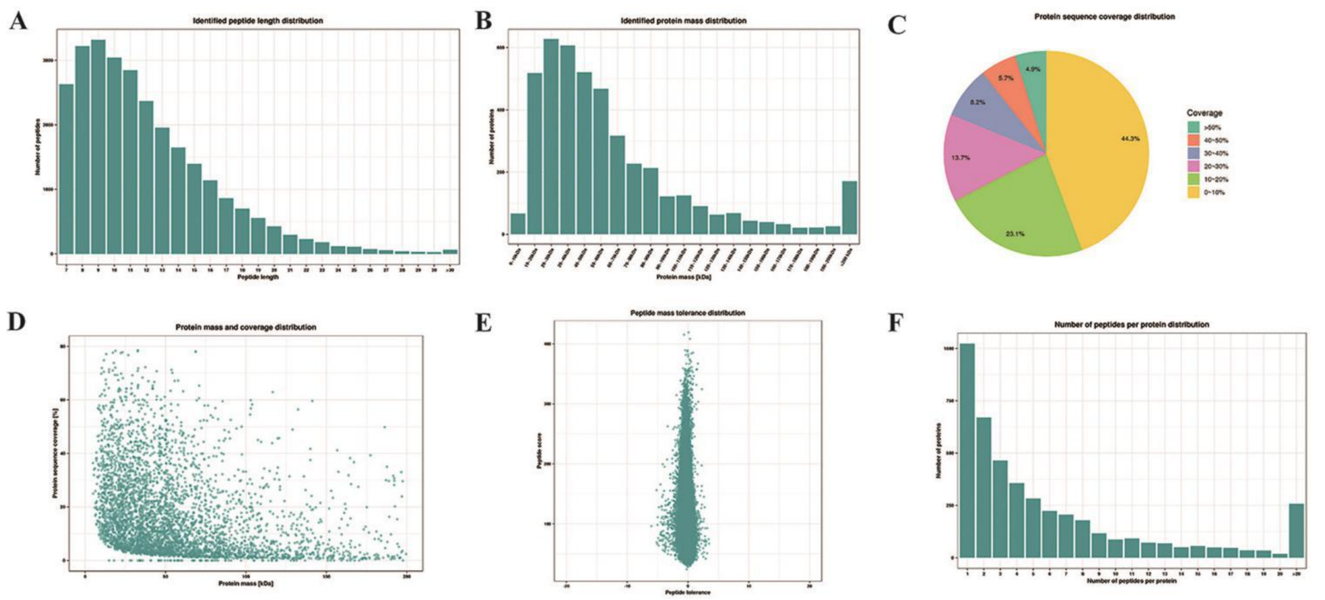

Supplementary Figure 3. Mass spectrometry quality control testing. (A) The length distribution of all identified peptides. (B) Molecular weight distribution of all identified proteins. (C) Protein coverage distribution. (D) The relationship between molecular weight and coverage of protein identified by mass spectrometry. (E) Mass accuracy distribution of mass spectrometer. (F) Number of peptides corresponding to each protein.

2 Supplementary Table

Supplementary Table 1. Primers for Real-time PCR

	Name	Sequences 5' to 3'
Cell	GAPDH F	GAAGACGGGCGGAGAGAAAC
	GAPDH R	GCCCAATACGACCAAATCCGT
	SM22α F	AAGCCTTCTTTCCCCAGACA
	SM22α R	TGCACTATGATCCACTCCACC
	BMP-2 F	CCCAGCGTGAAAAGAGAG
	BMP-2 R	CGTCCATTGAAAGAGCGT
	Runx2 F	GAGTGGACGAGGCAAGAGTT

	Runx2 R	GGATGAGGAATGCGCCCTAA
	TRAF6 F	TACTCATCAGAGAACAGATGCC
	TRAF6 R	TGTTCTCTTGTAGGTGGCGT
Rat	GAPDH F	AGTGCCAGCCTCGTCTCATA
	GAPDH R	AGAGAAGGCAGCCCTGGTAA
	SM22α F	AACGATGGACACTACCGTGG
	SM22α R	TTTGAAGGCCAATGACGTGC
	BMP-2 F	GACCCGCTGTCTTCTAGTGTT
	BMP-2 R	GGATAAGGGGCGCGATGC
	Runx2 F	CACAAGTGCGGTGCAAACTT
	Runx2 R	ATGACTCGGTTGGTCTCGGT
	TRAF6 F	CGCCAAAATGGAAACGCAGA
	TRAF6 R	TGCTTCCATCTCGGCAACTT
